# LRM-YOLO: A Lightweight YOLOv10n-Based Model for Forest Fire Smoke Detection in UAV Images

**DOI:** 10.3390/s26154887

**Published:** 2026-08-03

**Authors:** Yong Liu, Shaochen Jiang, Yongming Li, Jiajun Chen

**Affiliations:** College of Computer Science and Technology, XinJiang University, Urumqi 830046, China; 107552403819@stu.xju.edu.cn (Y.L.); lym@xju.edu.cn (Y.L.); 107552403772@stu.xju.edu.cn (J.C.)

**Keywords:** deep learning, forest fire smoke detection, LECD, lightweight network, MPDIoU, RepViTBlock, YOLOv10n

## Abstract

In recent years, unmanned aerial vehicles (UAVs) have gradually become an important tool for forest fire monitoring due to their flexibility and wide-area observation capability. However, existing detection algorithms still struggle to achieve a balance between computational complexity and detection performance in diverse background conditions, and data for such scenarios remain limited. Therefore, this paper proposes a lightweight forest fire smoke detection model based on YOLOv10n. Specifically, we introduce the RepViTBlock module to enhance smoke feature extraction and improve detection accuracy with low computational cost. Meanwhile, we design a Lightweight Efficient Convolutional Detection head (LECD), which improves smoke target recognition and localization while reducing the number of parameters and computational overhead of the detection head. We also adopt the Minimum Point Distance Intersection over Union (MPDIoU) as the bounding-box regression loss function to improve the localization accuracy of smoke bounding-box regression. In addition, we construct a UAV-perspective Forest Fire Smoke (UFFS) dataset, which contains typical forest fire smoke, nearby thin smoke, distant small-scale smoke, and smoke under diverse background conditions. Experiments were conducted on both the UFFS dataset and the Wildfire Smoke V1 dataset. The experimental results show that, compared with the baseline model, the proposed model reduces the number of parameters by 36.7% and GFLOPs by 42.3% on the UFFS dataset, while improving mAP50 by 1.3% and mAP50-95 by 3.7%. In addition, recall increases by 3.2% and precision increases by 3.6%, indicating an improved trade-off between detection accuracy and model complexity.

## 1. Introduction

Forest ecosystems play a crucial role in global climate regulation and biodiversity conservation, and they are also an important foundation for achieving ecological protection and carbon neutrality goals [1]. Under the influence of climate change and extreme weather, forest fires have become more sudden and destructive. They not only threaten human life and property but also release large amounts of pollutants. These pollutants can degrade air quality, endanger human health, and undermine ecosystem stability [2]. Previous studies have shown that timely fire detection can significantly shorten emergency response time and reduce ecological and economic losses. In the early stage of a fire, before flames have fully spread, smoke generated by incomplete combustion is usually the earliest observable precursor. Because smoke can spread over a wide area and persist for a relatively long period, smoke detection has become a key approach for early forest fire warning [3,4].

Existing forest fire monitoring technologies have mainly evolved through three stages: manual patrol, sensor-based monitoring, and vision-based detection [5]. Manual patrol is intuitive, but it is highly dependent on human experience and is limited in terms of coverage and timeliness. Sensor-based methods offer certain advantages in sensitivity, but they are often constrained by limited monitoring range as well as high deployment and maintenance costs. Forest fire monitoring can be conducted using satellites, ground cameras, and UAVs. Satellites are suitable for large-scale observation, but their ability to detect early-stage small-scale smoke is limited by spatial resolution and revisit frequency [6]. UAVs, with their flexibility, low-altitude operation, and high resolution, are more suitable for early smoke detection in forest patrol scenarios.

Different smoke detection methods exhibit distinct data adaptability. Traditional visual methods based on handcrafted color, texture, shape, motion, and spatiotemporal features are more suitable for fixed-camera videos with stable viewpoints and limited training data [7,8]. Satellite-based remote sensing covers vast geographical areas, yet its ability to continuously monitor a specific region is limited. Moreover, the generally coarse spatial resolution of satellite imagery makes it difficult to identify fire smoke that has not yet developed into a large-scale plume [9]. UAV-perspective images provide richer spatial details for small smoke plumes, and their flexibility enables multi-angle and multi-altitude maneuverable observations, while also introducing scale variation, viewpoint changes, and complex backgrounds [10,11].

In recent years, deep learning has significantly advanced visual smoke detection. Object detection methods are generally divided into two-stage and single-stage frameworks. Two-stage detectors, represented by R-CNN [12] and Faster R-CNN [13], provide strong feature extraction and localization capability, whereas single-stage detectors, such as SSD [14] and the YOLO series, are more suitable for real-time detection because of their higher inference efficiency. Early deep learning-based smoke detection studies demonstrated that convolutional neural networks can learn more discriminative smoke features than traditional handcrafted-feature methods [15,16]. More recently, YOLO-based detectors have become an important direction for forest fire smoke detection because of their favorable balance between detection accuracy and computational efficiency [17,18]. Wang et al. [19] proposed RT-DETR-Smoke, which employs an efficient hybrid encoder combining convolutional and Transformer features to preserve important smoke information while reducing computational cost. However, many accuracy-oriented improvements introduce additional modules, parameters, and computational overhead. Therefore, achieving a favorable balance between model complexity and smoke detection performance remains an important problem.

Bakirci [20] evaluated several YOLO variants for vehicle and vessel detection on a drone platform equipped with Jetson Xavier NX and found that YOLOv10n achieved a favorable balance among detection accuracy, inference speed, GPU utilization, and power consumption. The experimental results on the Jetson Xavier NX platform provide a practical basis for selecting YOLOv10n. Therefore, a lightweight forest fire smoke detection method for UAV imagery is of great significance and provides promising support for future UAV-based patrol scenarios. The main contributions of this study are summarized as follows:

- To support model training and evaluation, a UAV-perspective Forest Fire Smoke (UFFS) dataset is assembled, containing typical forest fire smoke, nearby thin smoke, distant small-scale smoke, and smoke under diverse background conditions.
- A RepViTBlock feature extraction module is introduced into the backbone network to enhance the extraction of weak-texture and multi-scale smoke features while maintaining relatively low computational cost.
- This paper proposes a lightweight and efficient convolutional detection head (LECD). Leveraging its inverted residual design, LECD reduces the number of parameters and computational overhead in the detection head while enhancing the ability to identify and localize smoke targets.
- MPDIoU is introduced as the bounding-box regression loss to improve the geometric constraints between predicted and annotated boxes. By incorporating corner-point distance constraints, MPDIoU provides localization constraints for smoke targets with blurred boundaries and irregular shapes, improving the localization accuracy of smoke bounding-box regression.

The remainder of this paper is organized as follows: Section 2 reviews related studies on smoke detection. Section 3 provides a detailed description of the specific modules of the proposed LRM-YOLO model, where “LRM” is derived from the initials of the three main improved components: LECD, RepViTBlock, and MPDIoU. Section 4 describes the experimental settings, datasets, evaluation metrics, and experimental results. Section 5 presents the discussion and conclusions of this study.

## 2. Related Work

### 2.1. Traditional Visual Smoke Detection Methods

Traditional forest fire smoke detection primarily relies on handcrafted features such as color, texture, motion, and spatiotemporal variations. Kikuta et al. [21] combined HSV color characteristics with optical-flow variance to distinguish smoke from other moving objects in outdoor videos, although the method was mainly designed for daytime fixed-camera monitoring. Hossain et al. [22] combined fire-specific color features, multi-color-space local binary patterns, and an artificial neural network to detect flames and smoke in UAV images. However, its performance remained dependent on manually designed color and texture features. In addition to improving visual feature design, some studies have considered the computational requirements of UAV-based forest fire monitoring. Üremek et al. [23] developed a low-power forest fire monitoring system that combines environmental sensors, LoRa communication, and machine-learning models for fire-risk assessment, although further validation is still needed. Li et al. [24] proposed a lightweight fire detection and segmentation model for a UAV-assisted mobile edge computing system, improving detection and warning efficiency while reducing the deployment burden on edge devices. Overall, these approaches improve smoke and fire monitoring through handcrafted visual features, environmental sensing, and resource-efficient system design. However, their performance often depends on predefined features, thresholds, or task-specific configurations. These limitations motivate the use of deep learning methods that can automatically learn discriminative smoke representations from complex scenes.

### 2.2. Monitoring Forest Fire Smoke Using UAVs and Deep Learning

With the rapid development of deep learning and UAV imaging technology, convolutional neural networks have been increasingly applied to forest fire smoke detection in aerial imagery, enabling automatic smoke feature learning and improved detection performance. Meanwhile, reducing model complexity while preserving detection accuracy has become an important concern in this field. Yang et al. [25] proposed an efficient forest fire smoke detection method for UAV imagery by improving YOLOv5 with K-means++ anchor clustering, partial convolution, a small-target detection head, coordinate attention, and transfer learning. The method improves small-smoke detection and inference efficiency; however, it still contains 11.1 M parameters and requires 13.3 GFLOPs, leaving room for further model compression. Chen et al. [26] proposed LMDFS, a lightweight YOLOv7-based model for forest fire smoke detection in UAV images. By incorporating GSConv, coordinate attention, CARAFE upsampling, and SIoU loss, LMDFS reduces the parameter count and improves small-smoke detection. Nevertheless, its computational cost remains relatively high at 25.1 GFLOPs despite having only 7.96 M parameters. Zhu et al. [27] proposed an improved YOLOv8 model for multiscale wildfire and smoke detection in complex UAV forest environments. The method modifies the C2F module and introduces EMA, AgentAttention, and BiFormer to strengthen contextual and multiscale feature extraction. Although it improves the detection of targets at different scales and viewing angles, its parameter count reaches 30.42 M, which increases the model storage and deployment requirements.

Overall, existing UAV-based forest fire smoke detection methods have improved small-target recognition and multiscale feature extraction through anchor optimization, lightweight convolution, attention mechanisms, and enhanced detection heads. However, reducing parameters and computational cost without weakening the representation and localization of small, low-contrast, and blurred-boundary smoke remains challenging. Therefore, further investigation of lightweight detection strategies that preserve reliable smoke detection performance is still warranted.

## 3. Proposed Method

### 3.1. Overall Framework

In this study, the YOLOv10n implementation provided by Ultralytics was adopted as the baseline model [28]. YOLOv10n is the nano variant of the YOLOv10 series and is commonly used as a representative real-time object detector due to its favorable inference speed and computational efficiency. To reduce model complexity while maintaining smoke detection performance, this study improves YOLOv10n from three aspects: backbone feature extraction, detection head design, and bounding-box regression supervision. These modifications aim to enhance the representation and localization of key smoke features while reducing the number of parameters and computational cost.

Specifically, RepViTBlock is introduced into the backbone because smoke regions in UAV-view images often contain weak texture and blurred boundaries, requiring more effective feature refinement under a lightweight structure. The lightweight efficient detection head, LECD, is designed because the detection head of YOLOv10n still accounts for a considerable proportion of parameters and computations, and reducing redundancy in this part can directly improve model efficiency. In addition, MPDIoU is adopted during training because corner-point distance constraints can provide additional geometric supervision for annotation-based smoke bounding-box regression, especially when smoke regions have irregular shapes and ambiguous visual boundaries. These improvements are integrated into a lightweight smoke detection framework named LRM-YOLO.

**Figure 1:**
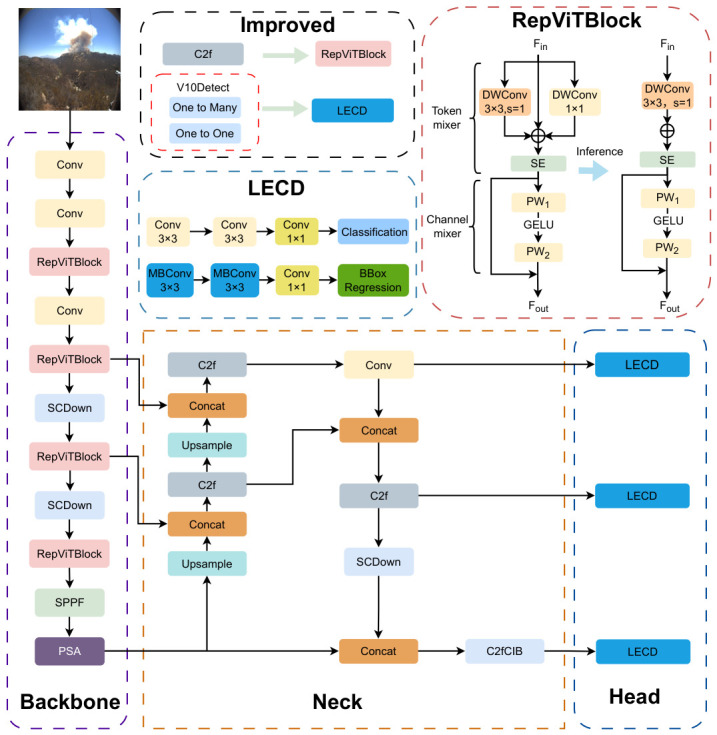
Diagram of the LRM-YOLO model architecture.

LRM-YOLO follows the Backbone–Neck–Head architecture of YOLOv10n. The Neck retains the Spatial Pyramid Pooling-Fast (SPPF) and Partial Self-Attention (PSA) modules of the baseline, while the main modifications are applied to the Backbone, Head, and training loss. Therefore, we propose the LRM-YOLO detection framework, whose overall network architecture is shown in Figure 1.

Although YOLOv10n possesses good real-time detection capabilities, it still suffers from insufficient representation of weak-feature smoke when applied to forest fire smoke detection tasks. A RepViTBlock is introduced into the Backbone to enhance the representation of smoke with blurred boundaries, small-scale details, and weak features; the Head employs our newly designed efficient detection head, LECD, to reduce the number of parameters and computational overhead while maintaining detection efficiency. Furthermore, during training, the MPDIoU loss function is adopted in place of the traditional regression loss to enhance the localization capability for targets with blurred boundaries and irregular shapes.

### 3.2. RepViTBlock Module

**Figure 2:**
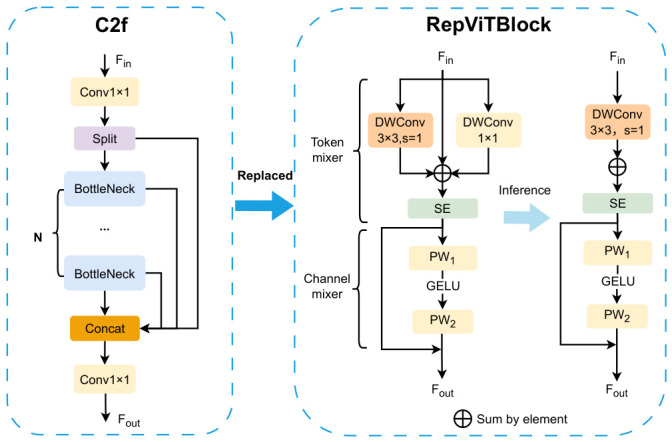
Replacement of C2f modules in the backbone with RepViTBlock.

RepViTBlock follows the design philosophy of RepViT, which separates token mixing and channel mixing in a MetaFormer-style manner. Specifically, spatial information interaction is mainly performed by the depthwise convolution-based token mixer, while inter-channel transformation is conducted by the pointwise-convolution-based channel mixer. In addition, structural re-parameterization is introduced into the token mixer during training to improve representation learning, and the multi-branch structure can be fused into a single-path structure during inference to reduce structural redundancy [29]. As shown in Figure 2, four C2f stages in the YOLOv10n backbone are replaced with RepViTBlock modules, while the original downsampling layers are retained to improve feature representation efficiency under lightweight constraints.

In the YOLOv10n configuration, RepViTBlock modules are introduced at four backbone stages, corresponding to layers 2, 4, 6, and 8. Under the nano-scale depth and width coefficients, these stages operate on feature maps at P2/4, P3/8, P4/16, and P5/32, with channel dimensions of 32, 64, 128, and 256, respectively. The corresponding effective repetition numbers are 1, 2, 2, and 1. Each RepViTBlock uses a stride of s=1 and preserves both the spatial resolution and channel dimension, while its hidden channel dimension is set to twice the input channel dimension. The original Conv and SCDown layers are retained between stages to perform spatial downsampling.

Based on this design, RepViTBlock separates spatial feature modeling from channel feature transformation. For an input feature map $F_{\mathrm{in}}\in R^{C\times H\times W}$, the corresponding output feature map $F_{\mathrm{out}}$ has the same dimensions because the input and output channel dimensions are identical and no downsampling is performed inside the block. Its overall forward process can be formulated as

(1) $$F_{\mathrm{out}}=C\left(T(F_{\mathrm{in}})\right)$$

where T(·) and C(·) denote the token mixer and channel mixer, respectively, while $F_{\mathrm{in}}$ and $F_{\mathrm{out}}$ represent the input and output feature maps. In this design, the token mixer is responsible for spatial information interaction, whereas the channel mixer performs inter-channel transformation and feature refinement.

When the stride is s=1, RepViTBlock is mainly used for feature enhancement while preserving the spatial resolution. The token mixer adopts a RepVGGDW-based structure, which can be expressed as:

(2) $$F_{t}=SE\left(DW_{3\times 3}\left(F_{\mathrm{in}}\right)+DW_{1\times 1}\left(F_{\mathrm{in}}\right)+F_{\mathrm{in}}\right)$$

where $DW_{3\times 3}\left(\cdot \right)$ and $DW_{1\times 1}\left(\cdot \right)$ denote depthwise convolutions, and SE(·) denotes the optional channel attention operation. In the implemented configuration, the token mixer contains a 3×3 depthwise convolution–batch-normalization branch, a 1×1 depthwise convolution–batch-normalization branch, an identity branch. The channel mixer then refines the features through pointwise transformation and residual connection:

(3) $$F_{\mathrm{out}}=F_{t}+PW_{2}\left(\delta (PW_{1}\left(F_{t}\right))\right)$$

where $PW_{1}\left(\cdot \right)$ and $PW_{2}\left(\cdot \right)$ denote pointwise convolutions, and δ(·) denotes the GELU activation function. The first 1×1 pointwise convolution expands the channel dimension from *C* to 2C, and the second projects it from 2C back to *C*. Both pointwise convolutions are followed by batch normalization. Since the channel mixer preserves the feature-map dimensions after projection, its output is added to $F_{t}$ through a residual connection.

The 3×3 depthwise convolution captures local spatial patterns within each channel, helping preserve the weak texture and diffuse boundary information of smoke, while the 1×1 depthwise and identity branches retain complementary and original features during training. The subsequent SE operation adaptively emphasizes channels containing informative smoke responses and suppresses less relevant background information. Moreover, applying RepViTBlock at the P2/4, P3/8, P4/16, and P5/32 backbone stages enables feature refinement at different spatial resolutions, supporting the representation of nearby large-scale smoke and distant small-scale smoke. After training, the parallel branches in RepVGGDW can be fused into an equivalent single 3×3 depthwise convolution, thereby simplifying the inference structure without changing its equivalent output.

### 3.3. Lightweight Efficient Convolutional Detection Head (LECD)

**Figure 3:**
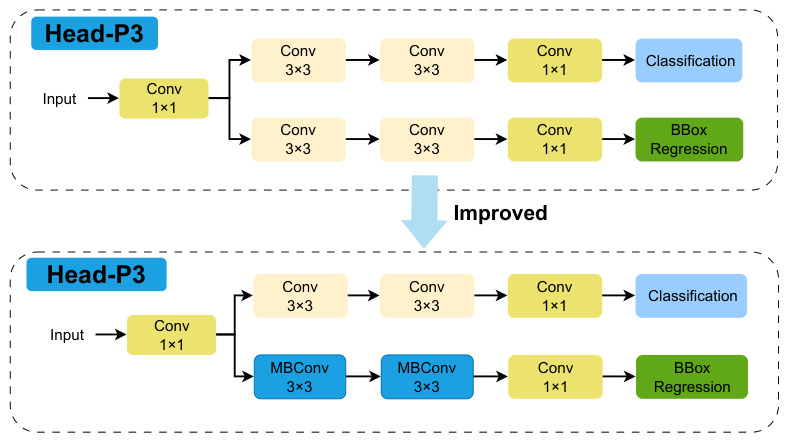
Structure of the LECD detection head.

The detection head is a critical component of object detection networks, as it is directly responsible for object classification and bounding-box regression. Its structural design has an important influence on the model’s ability to predict object categories, locations, and scale variations. To address parameter redundancy and insufficient computational efficiency in the baseline detection head [30], this study draws inspiration from the Mobile Inverted Bottleneck Convolution (MBConv) architecture in EfficientNet [31] and designs a lightweight and efficient detection head, termed LECD. The structure of the P3 detection head is illustrated in Figure 3. The LECD design is applied independently to the P3/8, P4/16, and P5/32 detection feature maps. At each scale, the bounding-box regression branch contains two consecutive MBConv blocks followed by a 1×1 prediction convolution, whereas the original lightweight classification branch is retained. In the proposed design, the regression branch is redesigned using MBConv-based modules, while the original lightweight classification branch is retained. This strategy avoids unnecessary modification of the entire prediction path and allows the optimization to focus more directly on the regression process, which is particularly important for smoke target localization.

In the implemented MBConv block, the expansion ratio is set to 1. Therefore, the optional 1×1 channel-expansion convolution is omitted. The input feature is directly processed by a 3×3 depthwise convolution with a stride of 1, followed by batch normalization and ReLU6 activation. The batch-normalization momentum and epsilon are set to 0.01 and $10^{-3}$, respectively. A 1×1 projection convolution followed by batch normalization is subsequently used to generate the output feature, without an additional activation after the projection layer.

Building on this structure, the Squeeze-and-Excitation module in the conventional MBConv design is replaced with the Convolutional Block Attention Module (CBAM) [32]. CBAM is enabled after the 3×3 depthwise convolution and before the 1×1 projection convolution in both MBConv blocks of each regression branch. It therefore operates on the multiscale semantic feature maps generated by the backbone and neck rather than directly on the input image.

**Figure 4:**
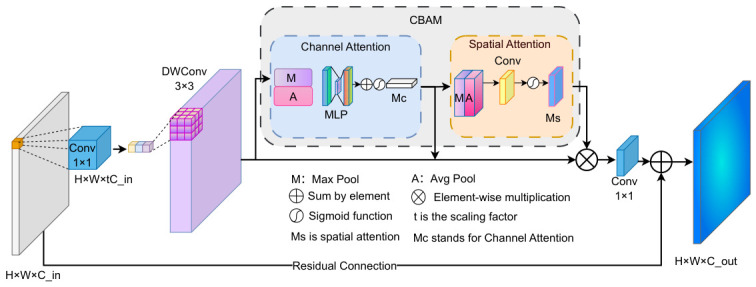
Architecture of the Improved MBConv Module.

The improved MBConv architecture is shown in Figure 4. In our experiments, this design improves the regression branch of the YOLOv10n baseline while reducing both the complexity and computational overhead of the detection head.

The channel-attention component of CBAM uses separate global-average-pooling and global-max-pooling branches with a channel reduction ratio of 16. Each branch contains a 1×1 convolution for channel reduction, a ReLU activation, another 1×1 convolution for channel restoration, and a sigmoid function. The two resulting channel-attention maps are added and multiplied by the input feature map. The spatial-attention component subsequently concatenates the channel-wise average and maximum feature maps and applies a 7×7 convolution followed by a sigmoid function to generate spatial attention weights. In this manner, channel-response recalibration is combined with spatial feature enhancement in the bounding-box regression branch. Residual connection is applied only when the stride is 1 and the input and output channel dimensions are identical, thereby preserving the original feature information and facilitating feature propagation.

### 3.4. MPDIoU Loss Function

**Figure 5:**
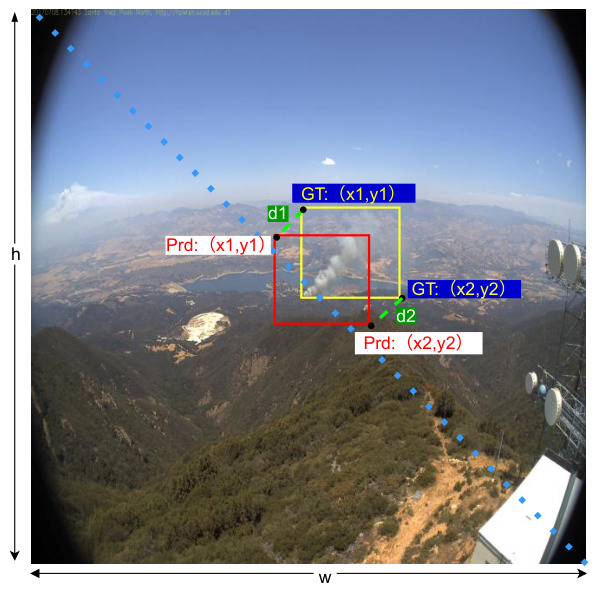
Schematic Diagram of the MPDIoU Principle. The dashed blue is the diagonal for $w^{2}+h^{2}$.

In forest fire smoke detection, this paper introduces the MPDIoU [33] loss function in the bounding-box regression stage to replace the Complete Intersection over Union (CIoU) regression loss used in the baseline model. Building upon the overlap-area constraint, MPDIoU further incorporates distance information between the keypoints of the predicted bounding box and the ground-truth bounding box. This allows it to explicitly describe the geometric deviation between the predicted box and the annotated box, thereby improving bounding-box regression accuracy under the adopted rectangular annotation protocol. It should be noted that this process aims to improve annotation-based localization rather than recover the exact physical boundary of smoke. MPDIoU extends IoU by introducing distance constraints between the corresponding points at the top-left and bottom-right corners of the predicted and ground-truth bounding boxes, defined as:

(4) $$L_{\mathrm{MPDIoU}}=1-\mathrm{IoU}+\frac{d_{1}^{2}}{w^{2}+h^{2}}+\frac{d_{2}^{2}}{w^{2}+h^{2}},$$

(5) $$d_{1}^{2}={\left(x_{1}^{p}-x_{1}^{g}\right)}^{2}+{\left(y_{1}^{p}-y_{1}^{g}\right)}^{2},$$

(6) $$d_{2}^{2}={\left(x_{2}^{p}-x_{2}^{g}\right)}^{2}+{\left(y_{2}^{p}-y_{2}^{g}\right)}^{2}.$$

where $d_{1}$ and $d_{2}$ represent the Euclidean distances between the corresponding corner points of the two boxes. Specifically, $\left(x_{1}^{p},y_{1}^{p}\right)$ and $\left(x_{2}^{p},y_{2}^{p}\right)$ denote the coordinates of the top-left and bottom-right corners of the predicted box, respectively, while $\left(x_{1}^{g},y_{1}^{g}\right)$ and $\left(x_{2}^{g},y_{2}^{g}\right)$ denote the coordinates of the top-left and bottom-right corners of the ground-truth bounding box, respectively. The MPDIoU loss function is illustrated in Figure 5.

In this study, the ground-truth bounding box is used as the regression target to supervise bounding-box prediction during training. Specifically, for each input image, the detection head predicts a bounding-box, and the MPDIoU loss is computed between the two bounding boxes to quantify their geometric discrepancy. This loss is then backpropagated through the network, and the model parameters are updated by gradient-based optimization, so that the predicted box is gradually guided toward the annotated smoke region. Since a rectangular bounding box can be represented by its top-left and bottom-right corner points, MPDIoU uses the distances between corresponding corner points to describe the overall deviation between the predicted and annotated boxes. Therefore, in our work, MPDIoU improves the accuracy of annotation-based bounding box regression rather than recovering the exact physical boundary of diffuse smoke.

Based on this annotation-based regression mechanism, MPDIoU enhances the localization accuracy of predicted bounding boxes. This is useful for diffuse smoke detection, where the annotated box provides an approximate but practical representation of the visually distinguishable smoke region. Under this annotation protocol, MPDIoU serves as an regression constraint for improving box localization quality, while introducing almost no additional computational cost [33].

## 4. Experiments and Results

### 4.1. Datasets

**Figure 6:**
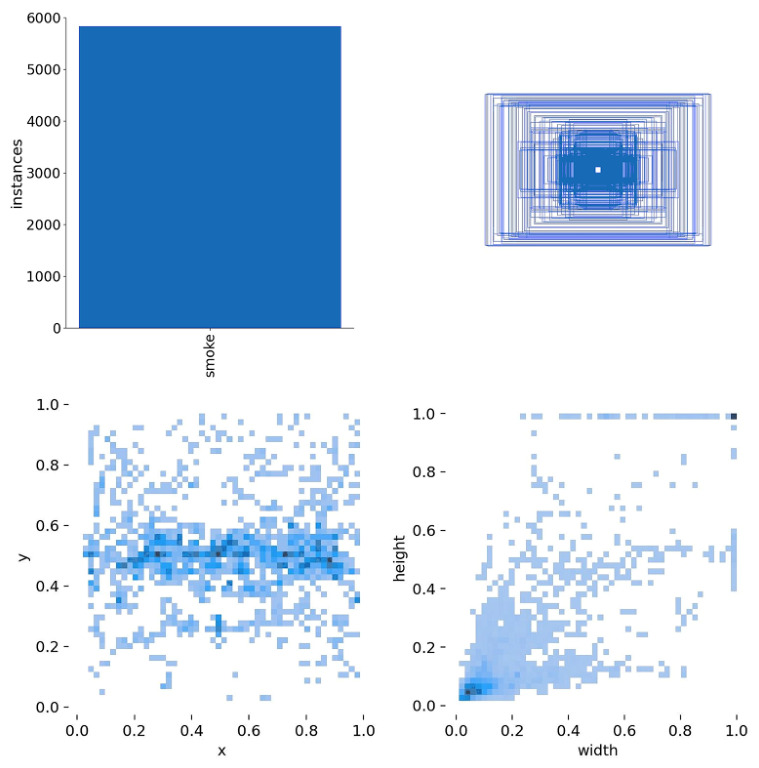
Statistical characteristics of the UFFS dataset.

Deep learning-based detection performance is closely related to the scale and diversity of training data. However, annotated forest fire smoke images with UAV-view or UAV-like aerial perspectives remain relatively limited. Therefore, we constructed the UFFS dataset by collecting and screening publicly available online static images with high-angle, top-down, or UAV-view aerial characteristics. UFFS contains 5012 smoke-positive images, covering typical forest fire smoke, nearby thin smoke, distant small-scale smoke, and smoke under diverse background conditions. The dataset includes 5833 annotated smoke bounding boxes. All images were resized to 640 × 640 during model preprocessing. Smoke instances were divided into three categories according to the ratio of the annotated bounding-box area to the total image area: small (<1%), medium (1–10%), and large (≥10%). Small, medium, and large smoke instances accounted for 45.8%, 36.5%, and 17.7% of all annotated instances, respectively. The resolution distribution of images, the number of annotated bounding boxes per image, and the smoke size distribution in the UFFS dataset are illustrated in Figure 6.

To further validate the proposed model, experiments were also conducted on the Wildfire Smoke V1 dataset [34], which contains 744 annotated smoke images. Compared with UFFS, this dataset generally contains more visually salient smoke targets and relatively limited variations in scene and viewpoint. It is therefore used to evaluate the model’s detection performance on other datasets.

To ensure dataset relevance and annotation consistency, explicit image selection and annotation rules were adopted during dataset construction. Only images containing visible smoke in forest, mountain, grassland, wildland, or other natural outdoor environments were retained. Images that were irrelevant to forest or wildland smoke detection, severely blurred, low-resolution, dominated by flames without clear smoke regions, or affected by prominent watermarks were excluded. Before dataset splitting, we performed a duplicate screening using 64-bit perceptual hashing (pHash). Image pairs with a Hamming distance no greater than 4 were treated as potential near duplicates and manually inspected. Obvious duplicate or nearly identical images were removed before splitting to reduce sample redundancy and potential overlap among subsets. The annotation rules of the UFFS dataset are summarized as follows:

- All smoke regions were annotated using Roboflow (available online: https://roboflow.com; accessed on 15 March 2025) and exported in YOLO format.
- The target was defined as the visually distinguishable smoke plume region, rather than the entire potential diffusion area.
- For diffuse smoke with ambiguous boundaries, only the main visible smoke region was tightly enclosed, while background regions such as clouds, fog, mountains, vegetation, and sky regions were avoided as much as possible.
- If multiple independent smoke regions appeared in one image, each region was annotated as a separate object.
- Ambiguous and challenging annotations were manually reviewed and corrected to ensure annotation consistency and quality.

**Figure 7:**
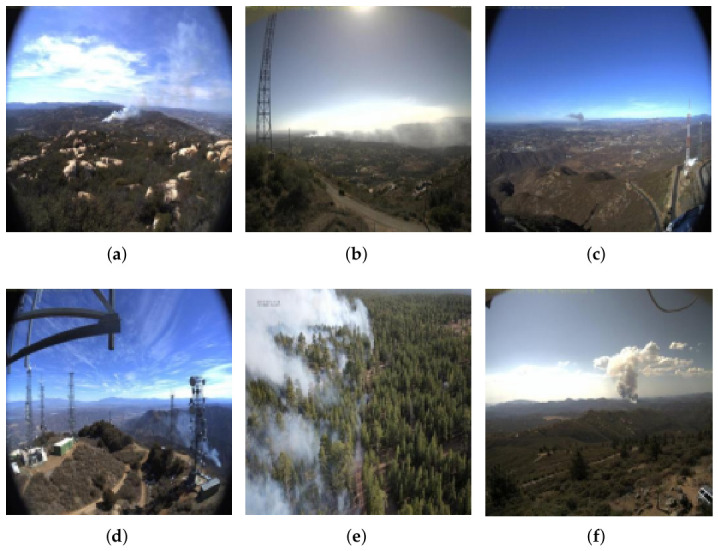
Typical smoke samples in the dataset: (**a**) Typical smoke. (**b**) Thin smoke under backlighting conditions. (**c**) Small-scale smoke. (**d**) Occluded smoke captured at the image edge. (**e**) Smoke captured from a UAV perspective. (**f**) Smoke under a complex and cloudy background.

To ensure scientific rigor and fairness, the final dataset was divided into training, validation, and test sets at a fixed ratio of 8:1:1. The training set was used for model parameter learning, the validation set for hyperparameter tuning and training monitoring, and the test set for final performance evaluation. The specific dataset split and ratio are shown in Table 1. To illustrate the diversity of the UFFS dataset, Figure 7 presents several representative smoke images from UAV perspectives under different environmental conditions, covering variations in smoke scale, illumination, occlusions, viewing angle, and background. These examples show the range of smoke appearances and imaging conditions considered in this dataset.

### 4.2. Experimental Environment and Evaluation Metrics

#### 4.2.1. Experimental Environment

All experiments were conducted using the PyTorch deep learning framework, and the proposed model was implemented based on the YOLOv10 framework provided by Ultralytics [28]. To improve reproducibility, the exact YOLOv10 implementation, Ultralytics version, and model configuration files were kept consistent across all experiments. The proposed model was initialized using the pre-trained weights of yolov10n.pt. All comparison models were trained and evaluated under the same dataset split, input image size, training epochs, optimizer settings, and evaluation protocol to ensure a fair comparison.

These strategies included color perturbations based on Hue, Saturation, and Value (HSV) transformation, geometric transformations such as random translation and scaling, and mosaic augmentation, which helped the model recognize smoke targets with different scales and morphologies. Additionally, a random erasure method was introduced to improve the model’s adaptability to local occlusions and background interference, thereby improving detection performance for smoke targets with weak textures and low contrast. The specific configuration is shown in Table 2.

The main training settings and hyperparameters used in the experiments are summarized in Table 3.

#### 4.2.2. Evaluation Metrics

The detection performance of the model is evaluated using Precision, Recall, mAP50, and mAP50-95. Model lightweightness and inference efficiency are further assessed using the number of parameters (Params), computational complexity (GFLOPs), and inference speed (FPS). Specifically, Precision reflects the proportion of correctly detected smoke targets among all predicted targets, whereas Recall reflects the model’s ability to identify actual smoke targets. mAP50 and mAP50-95 are used to comprehensively evaluate the overall detection performance of the model. Params and GFLOPs characterize model size and computational cost, respectively. FPS serves as an auxiliary indicator for comparing the forward inference efficiency of different models under identical experimental settings. It should be noted that the FPS reported in this study is computed only from the forward inference time of the model and does not include image preprocessing or post-processing time. The calculation methods of these evaluation metrics are presented below.

1. Precision: Precision measures the proportion of correctly predicted positive samples among all predicted positive samples, reflecting the prediction accuracy of the model.
  (7) $$\mathrm{Precision}=\frac{TP}{TP+FP}$$
  where TP denotes the number of true positives, i.e., positive samples correctly predicted as positive, and FP denotes the number of false positives, i.e., negative samples incorrectly predicted as positive.
2. Recall: This metric measures the proportion of correctly predicted positive samples among all actual positive samples, reflecting the detection completeness of the model.
  (8) $$\mathrm{Recall}=\frac{TP}{TP+FN}$$
3. mAP50 and mAP50-95: Mean Average Precision (mAP) is a widely used metric for evaluating object detection performance. In this study, mAP50 and mAP50-95 are used to evaluate the detection accuracy of the model. mAP50 denotes the mean Average Precision when the Intersection over Union (IoU) threshold is set to 0.5, while mAP50-95 denotes the mean Average Precision averaged over multiple IoU thresholds from 0.5 to 0.95 with a step size of 0.05.
  (9) $${\mathrm{AP}}_{t}=\int_{0}^{1}P_{t}\left(R\right)\;dR$$
  (10) $$\mathrm{mAP}50=\frac{1}{N}\sum_{i=1}^{N}AP_{i,0.50}$$
  (11) $$\mathrm{mAP}50\text{-}95=\frac{1}{10N}\sum_{t\in \{0.50,0.55,\ldots ,0.95\}}\sum_{i=1}^{N}AP_{i,t}$$
  where $P_{t}\left(R\right)$ represents the precision as a function of recall under the IoU threshold *t*, *R* denotes recall, $AP_{i,t}$ denotes the Average Precision of the *i*-th category at the IoU threshold *t*, and *N* denotes the total number of categories.
4. GFLOPs: GFLOPs denotes the number of floating-point operations required by the model, measured in billions, and reflects computational complexity.
  (12) $$\mathrm{GFLOPs}=\frac{\mathrm{FLOPs}}{10^{9}}$$
5. Parameters: Parameters represent the total number of learnable weights in the model, reflecting the model size and storage cost.
  (13) $$\mathrm{Parameters}=\sum_{l=1}^{L}\left(W_{l}+B_{l}\right)$$
  where $W_{l}$ and $B_{l}$ denote the weights and biases of the *l*-th layer, respectively, and *L* denotes the total number of layers.
6. FPS: Frames per second indicates the number of images processed by the model per second, reflecting its real-time inference capability.
  (14) $$\mathrm{FPS}=\frac{1000}{T}$$
  where *T* denotes the average forward inference time per image (ms).

### 4.3. Ablation Experiment

An ablation study was conducted to evaluate the contribution of each module in the final model. To address the challenge of balancing model complexity and detection performance in UAV-perspective wildfire smoke detection, YOLOv10n was adopted as the baseline model and a stepwise module-adding strategy was employed under consistent dataset partitioning, training settings, and experimental conditions. The individual and combined effects of RepViTBlock, LECD, and MPDIoU were systematically analyzed. By comparing different model combinations in terms of Precision, Recall, mAP50, mAP50-95, parameter count, GFLOPs, and FPS, we evaluated the contribution of each module to detection accuracy, lightweight design, and inference capability. As presented in Table 4, the proposed modules show different effects on detection metrics and model complexity.

As shown in Table 4, different modules contribute to different aspects of the model. When RepViTBlock is introduced alone, the number of parameters decreases from 2.691 M to 2.423 M, and the GFLOPs decrease from 8.22 to 7.64. Precision, mAP50, and mAP50-95 also show slight increases compared with the YOLOv10n baseline. Although Recall decreases slightly from 86.9% to 85.1%, this reduction is acceptable in the lightweight trade-off because RepViTBlock reduces model complexity while maintaining comparable overall detection performance. Among the three modules, LECD shows the most evident contribution to both detection performance and lightweight design. Compared with the baseline, introducing LECD alone increases Precision from 89.2% to 91.6%, Recall from 86.9% to 90.1%, mAP50 from 93.8% to 94.8%, and mAP50-95 from 64.5% to 67.4%. Meanwhile, the parameter count decreases from 2.691 M to 1.701 M, the GFLOPs decrease from 8.22 to 4.72, and the FPS increases from 357 to 833. These results indicate that LECD plays a major role in reducing the detection-head complexity while improving detection-related metrics. When MPDIoU is used alone, the model complexity remains unchanged because it modifies only the bounding-box regression loss and does not introduce additional inference-time structures. Accordingly, its parameter count and GFLOPs remain 2.691 M and 8.22, respectively. Compared with the baseline, MPDIoU improves Recall, mAP50, and mAP50-95 from 86.9%, 93.8%, and 64.5% to 88.4%, 94.1%, and 64.7%, respectively. This indicates that MPDIoU provides additional localization constraints for bounding-box regression, although its individual contribution is mainly reflected in moderate improvements in localization-related metrics. When the three modules are combined, the final model achieves 92.8% Precision, 90.1% Recall, 95.1% mAP50, and 68.2% mAP50-95, with 1.704 M parameters and 4.74 GFLOPs. Although its FPS is lower than that of the LECD-only configuration, it remains higher than that of the baseline. Overall, the ablation results indicate that the final configuration provides a balanced trade-off among detection performance, model complexity, and inference speed.

Additionally, for our proposed LECD, we present ablation results for the two attention modules (SE and CBAM). Compared with SE, CBAM introduces only a small increase in the number of parameters and GFLOPs. Nevertheless, it shows overall performance improvement in the smoke detection task. By combining channel and spatial attention, CBAM enables the model to better capture features of smoke regions with weak textures, blurred boundaries, and diffuse visual patterns. It improves detection performance while still satisfying lightweight design requirements. The results are shown in Table 5.

### 4.4. Comparative Experiment of Different Lightweight Models

As shown in Table 6, LRM-YOLO improves the detection performance of the YOLOv10n baseline while reducing model complexity. Several lightweight detection models, including YOLOv5n [35], YOLOv6 [36], YOLOv7-tiny [37], YOLOv8n [38], YOLOv10n [28], YOLOv10s [28], YOLOv11n [39], YOLOv12n [40], YOLOv13n [41], and YOLO26n [42], are included as reference models for comparison under the same dataset and evaluation protocol. The results show that LRM-YOLO achieves improved mAP50 and lower model complexity compared with the YOLOv10n baseline. To ensure a fair comparison among reproducible lightweight detection models, all YOLO-based models were trained and evaluated under the same experimental protocol, including the same UFFS dataset split, input image size, training epochs, optimizer settings, and evaluation metrics. Official pretrained weights were used for initialization where available.

Compared with the YOLOv10n baseline, LRM-YOLO improves mAP50 from 93.8% to 95.1% and mAP50-95 from 64.5% to 68.2%, while reducing the number of parameters from 2.691 M to 1.704 M and GFLOPs from 8.22 to 4.74.

This indicates that the proposed modifications improve the accuracy–complexity trade-off of the YOLOv10n baseline for forest fire smoke detection using UAV imagery. In general, the proposed method reduces the number of parameters and computational overhead of the YOLOv10n baseline while improving its forest fire smoke detection performance on the evaluated datasets. The diverse scenes included in the UFFS dataset also provide a range of conditions for model evaluation, under which the proposed model exhibits relatively stable performance.

### 4.5. Evaluation on the Wildfire Smoke V1 Dataset

To further examine the performance of the proposed model on different datasets, we conducted additional experiments on the public Wildfire Smoke V1 dataset. In this experiment, both the baseline and our model were trained exclusively on the UFFS training set and then evaluated directly on the Wildfire Smoke V1 dataset. No retraining, fine-tuning, or parameter tuning was performed on the Wildfire Smoke V1 dataset. The corresponding results are shown in Table 7.

As shown in Table 7, the evaluation results on the Wildfire Smoke V1 dataset indicate that LRM-YOLO achieves slightly improved detection performance compared with the YOLOv10n baseline while maintaining lower model complexity. Specifically, Precision and mAP50-95 showed moderate gains, whereas Recall and mAP50 achieved larger numerical increases. These results indicate that, under direct evaluation without retraining or fine-tuning, LRM-YOLO maintains better evaluation metrics than the YOLOv10n baseline on the Wildfire Smoke V1 dataset.

### 4.6. Visualization Results and Analysis

#### 4.6.1. Visualization of Detection Results

To further validate the effectiveness of the proposed method, this paper visually compares the detection results of the improved model with those of the baseline YOLOv10n, including the ground-truth bounding boxes, the predictions of the baseline model, and those of the improved model.

**Figure 8:**
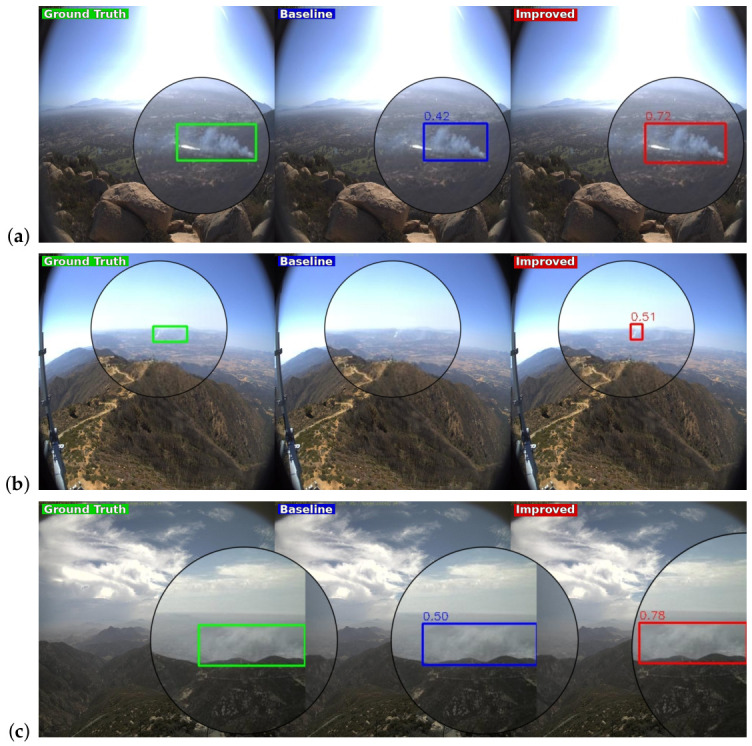
Comparison of test results: (**a**) The baseline fails to detect small targets, whereas the improved model can detect them accurately. (**b**) The improved model produces more accurate bounding boxes and confidence scores than the baseline. (**c**) For thin smoke, the improved model delivers more accurate detection results than the baseline model.

As shown in Figure 8, the figure presents a comparison of forest fire smoke detection results between the improved model and the YOLOv10n baseline under diverse background conditions. In Figure 8a, the baseline YOLOv10n fails to detect small smoke targets, whereas the improved model can identify them accurately. In Figure 8b, the improved model produces more accurate bounding boxes and higher confidence scores than the baseline. In Figure 8c, for thin smoke targets, the improved model also achieves more reliable detection results.

Overall, in the presented UAV-perspective forest smoke images, LRM-YOLO detects several weak and small-scale smoke regions that are missed by the YOLOv10n baseline, and its predicted bounding boxes are more tightly aligned with the visible smoke areas. Under backlighting and cloud-interference conditions, LRM-YOLO also provides more stable confidence scores. Across examples with different backgrounds, the proposed method achieves better detection and localization of visible smoke regions than the YOLOv10n baseline, preliminarily indicating improvements in feature recognition and localization capability.

#### 4.6.2. Heatmap Visualization Analysis

**Figure 9:**
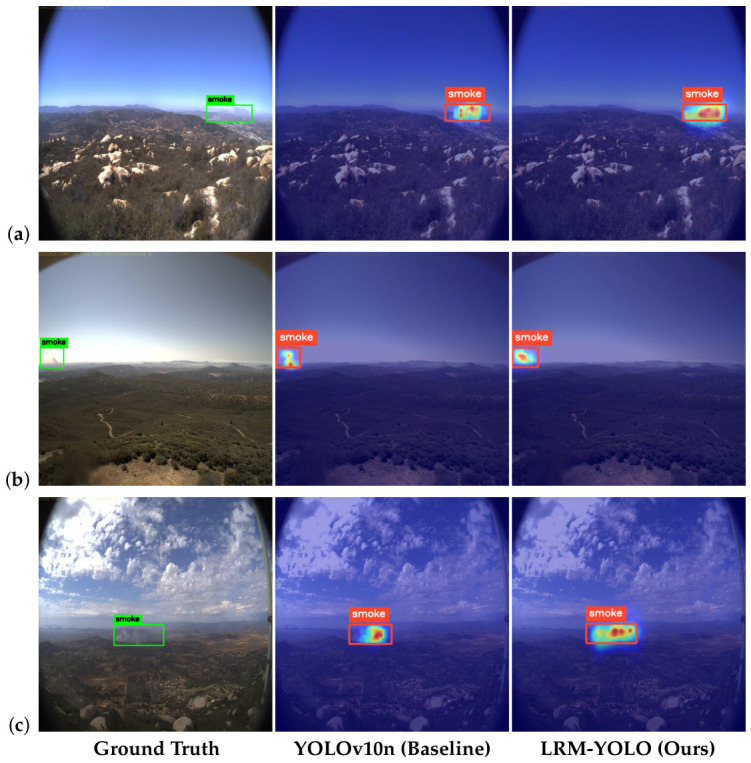
Comparison results of Grad-CAM heatmaps between the baseline and the proposed model: (**a**) Smoke under normal conditions. (**b**) Smoke in backlighting scenes. (**c**) Smoke under cloudy conditions.

Gradient-weighted Class Activation Mapping (Grad-CAM) generates spatial activation maps based on feature contributions to a target prediction, helping determine whether the detector focuses on target regions or irrelevant backgrounds. To examine the spatial regions involved in the model predictions, Grad-CAM was applied to YOLOv10n and LRM-YOLO. Representative samples under normal conditions, backlighting conditions, and cloud-background conditions were selected for visualization. Figure 9 presents the corresponding ground-truth annotations and activation maps, where warmer regions indicate stronger contributions to the predicted smoke detections.

The Grad-CAM heatmaps were used to visualize and compare the smoke-related feature responses of YOLOv10n and LRM-YOLO. In the heatmaps, red and yellow regions indicate stronger activation and higher contribution to smoke detection, whereas green and blue regions indicate weaker activation. As shown in Figure 9a, both models can attend to the clearly visible smoke region under normal conditions, while LRM-YOLO shows a more concentrated response around the main smoke area. In Figure 9b, although the smoke target is weak under backlighting conditions, the proposed model still maintains visible activation within the smoke region. In Figure 9c, where smoke appears against a cloudy background, LRM-YOLO also shows stronger activation in the smoke region than the baseline. Overall, these visualizations suggest that the proposed lightweight model can maintain smoke-related activation more consistently under normal, backlighting, and cloudy-background conditions.

#### 4.6.3. Training Curves Presentation

**Figure 10:**
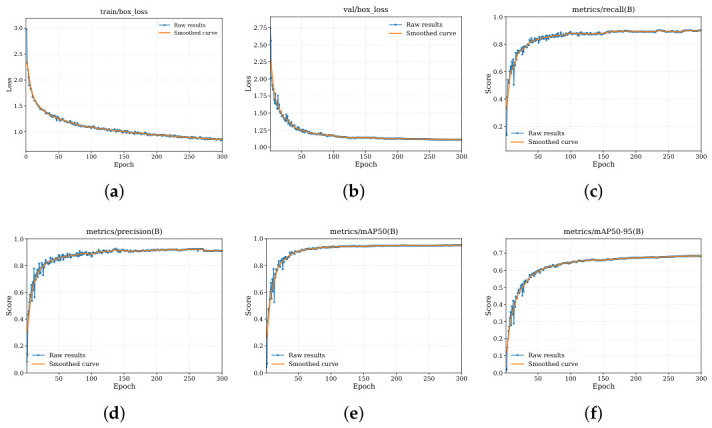
Training and evaluation curves of the proposed model: (**a**) training box loss; (**b**) validation box loss; (**c**) recall curve; (**d**) precision curve; (**e**) mAP50 curve; (**f**) mAP50-95 curve.

Figure 10 presents the training dynamics of the proposed model, including the training and validation losses, Precision, Recall, mAP50, and mAP50-95. As training progresses, both the training and validation losses show a continuous downward trend and gradually converge, indicating that the model learns the smoke-related features without obvious instability. Meanwhile, the evaluation metrics increase rapidly in the early training stage and then gradually stabilize. The steady improvement and final convergence of Precision, Recall, mAP50, and mAP50-95 demonstrate that the model achieves stable detection performance.

#### 4.6.4. Hard Positive Evaluation

To further evaluate the detection performance of the models for small-scale smoke targets under challenging positive conditions, we manually screened small-smoke samples from the original test set. A sample was included when the area of its annotated smoke bounding box accounted for less than 1% of the total image area. Based on this criterion, a challenging positive small-smoke test subset was constructed. The subset contains 178 UAV-view smoke images and 178 annotated smoke instances, with each image containing one smoke target. All samples were selected only from the original test set and were used exclusively for additional evaluation; they were not involved in model training, validation, hyperparameter tuning, or model selection. According to their scene characteristics, the samples were divided into three categories: small smoke against cloudy backgrounds, small smoke under backlighting conditions, and thin or visually weak small smoke. The detailed composition of the subset is presented in Table 8.

These samples are characterized by small target scales, diffuse smoke boundaries, and low contrast between the smoke targets and their surrounding backgrounds. Such characteristics may increase the likelihood of missed detections and false detections. Therefore, Precision, Recall, F1-score, the number of missed detections, the number of false detections, miss rate, false positives per image (FPPI) and mAP50 were calculated to provide a more comprehensive evaluation of the models under challenging small-smoke conditions.

The miss rate and FPPI are calculated as follows:

(15) $$\mathrm{Miss}\;\mathrm{Rate}=\frac{\mathrm{FN}}{\mathrm{GT}}\times 100\%,$$

(16) $$\mathrm{FPPI}=\frac{\mathrm{FP}}{N_{\mathrm{img}}},$$

where FN denotes the number of missed smoke instances, FP denotes the number of false detections, GT denotes the total number of ground-truth smoke instances, and $N_{\mathrm{img}}$ denotes the total number of evaluated images. The experimental results are reported in Table 9.

As shown in Table 9, LRM-YOLO detects 171 of the 178 annotated smoke instances, compared with 166 instances detected by YOLOv10n. Accordingly, the number of missed detections decreases from 12 to 7, corresponding to a reduction of five missed instances. The miss rate decreases from 6.74% to 3.93%, while Recall increases from 93.26% to 96.07%. These results indicate that the proposed model misses fewer small smoke instances in the evaluated images.

LRM-YOLO also reduces the number of false detections from 28 to 22. Precision increases by 3.03 percentage points, from 85.57% to 88.60%, and the F1-score increases from 89.25% to 92.18%. Meanwhile, FPPI decreases from 0.157 to 0.124 false detections per image. This tendency suggests that the reduction in missed detections is not obtained by producing more false-positive predictions; instead, the proposed model shows a more balanced Precision–Recall result at the adopted confidence and IoU thresholds. The mAP50 values of the two models are relatively close, increasing from 95.19% for YOLOv10n to 96.30% for LRM-YOLO. Compared with this limited difference in the threshold-averaged metric, the changes in FN, FP, Recall, miss rate, and F1-score are more apparent at the selected operating point. Overall, the results suggest that the proposed modifications may help reduce missed and false detections for small smoke targets occupying less than 1% of the image area in the evaluated challenging scenes.

#### 4.6.5. Failure Case Analysis

**Figure 11:**
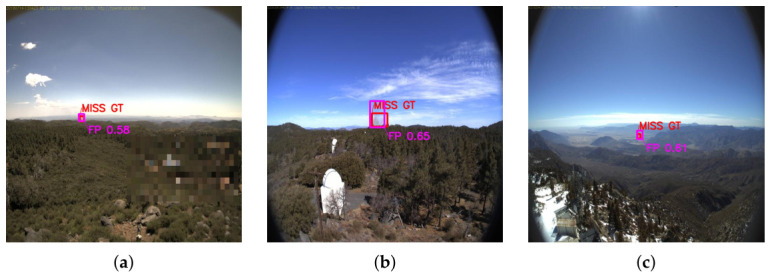
Failure cases of LRM-YOLO on the challenging positive small-smoke subset. In (**a**–**c**), small smoke targets under backlighting conditions exhibit weak visual features and low contrast with the surrounding background, resulting in missed detections and false-positive predictions.

The quantitative results in the previous section show that LRM-YOLO produces fewer missed and false detections than the YOLOv10n baseline on the hard positive small-smoke subset, although some detection errors still remain. To further investigate the performance boundary of the model under extreme conditions, Figure 11 presents representative failure cases of LRM-YOLO on this subset.

In these three failure cases, the smoke targets occupy only a very small image area and appear under backlighting conditions, resulting in weak contrast and limited visible smoke features. Under such conditions, the model may fail to generate a correct response to the actual smoke target, leading to missed detections. At the same time, bright or diffuse background regions with smoke-like appearances may be incorrectly identified as smoke, resulting in false-positive predictions. These errors become more likely when several unfavorable visual factors occur simultaneously, including small target size, backlighting, weak smoke appearance, and visually similar background regions. The presented cases indicate that the proposed model still has difficulty detecting very weak small smoke and distinguishing it from smoke-like background interference under such combined conditions. Although these cases reveal the remaining limitations of the model, they occur under relatively uncommon and particularly difficult background conditions. Within the scope of this lightweight detection study, the overall results in Table 9 still show that LRM-YOLO produces fewer missed detections and false-positive predictions than the YOLOv10n baseline.

## 5. Discussion and Conclusions

### 5.1. Discussion

A key finding of this study is that LRM-YOLO improves the accuracy–complexity trade-off of the YOLOv10n baseline on the evaluated smoke detection datasets. The results show that the proposed structural modifications reduce the number of parameters and computational overhead while maintaining or improving detection performance. This suggests that model efficiency can be improved without simply increasing model scale.

This improvement is further supported by the ablation results of the three proposed components. RepViTBlock, LECD, and MPDIoU contribute to backbone feature representation, detection-head efficiency, and bounding-box regression, respectively. Despite these improvements, the proposed model still has several limitations. First, although the additional end-to-end FPS test shows that LRM-YOLO achieves 87 FPS with a batch size of 1 on an NVIDIA GeForce RTX 3090 Ti GPU, this result was obtained under a desktop-GPU setting. Therefore, it should be regarded as a practical inference-efficiency reference rather than direct evidence of onboard UAV deployment performance. Further tests on embedded UAV platforms are still required to evaluate latency stability, power consumption, and real-time performance under actual deployment conditions.

Second, although the current dataset contains UAV-view images under multiple scene conditions, it still cannot fully cover the complexity of real UAV-based forest monitoring scenarios. UFFS mainly focuses on smoke-positive images and contains relatively limited hard negative samples. In practical UAV patrols, imaging angle, flight altitude, viewing distance, camera motion, illumination, terrain occlusion, and background composition may vary substantially. In addition, fog, mist, haze, dust, steam, and thin clouds may exhibit low contrast, diffuse boundaries, and semi-transparent visual patterns similar to early-stage smoke. These smoke-like interferences may still lead to false-positive predictions under broader UAV imaging conditions.

Third, although the challenging positive small-smoke subset shows that LRM-YOLO reduces missed detections and false-positive predictions for small smoke targets, this evaluation is still limited to positive images containing annotated smoke instances. It does not fully represent smoke-free scenes or complex hard-negative backgrounds. In addition, some smoke targets may still be missed when they are extremely weak, very small, heavily occluded, or visually indistinguishable from the surrounding background.

### 5.2. Conclusions

This paper proposes LRM-YOLO, a lightweight forest fire smoke detection model based on YOLOv10n. The model improves the original YOLOv10n framework from three aspects, including backbone feature extraction, detection-head design, and bounding-box regression. The aim is to improve the balance between model complexity and detection performance for UAV-view forest fire smoke detection.

Experiments on the UFFS dataset show that LRM-YOLO improves mAP50 from 93.8% to 95.1% and mAP50-95 from 64.5% to 68.2% compared with YOLOv10n. Precision increases from 89.2% to 92.8%, while the number of parameters decreases from 2.691 M to 1.704 M and GFLOPs decrease from 8.22 to 4.74. These results correspond to reductions of approximately 36.7% and 42.3% in parameters and GFLOPs, respectively. On the Wildfire Smoke V1 dataset, LRM-YOLO also maintains better evaluation metrics than YOLOv10n under direct evaluation without retraining or fine-tuning.

The ablation experiments further show that RepViTBlock, LECD, and MPDIoU each contribute to the final model performance. In addition, the detection visualizations and Grad-CAM heatmaps indicate that, in the presented examples, LRM-YOLO produces more reliable responses than the baseline under normal smoke conditions, backlighting scenes, and cloudy backgrounds. The challenging positive small-smoke evaluation also shows fewer missed detections and fewer false-positive predictions at the selected confidence and IoU thresholds. These results provide additional evidence that the proposed model improves smoke detection performance while reducing model complexity.

Overall, LRM-YOLO provides a lightweight detection approach for forest fire smoke detection under the evaluated conditions. However, broader validation is still needed in real UAV monitoring scenarios with more complex scenes, hard negative samples, and onboard deployment constraints. Future work will focus on expanding hard-negative samples, evaluating the model under broader UAV imaging conditions, and testing its performance on embedded UAV platforms.

## Figures and Tables

**Table 1:** Details of the dataset split.

Datasets	Train	Valid	Test	Total
UFFS	4010	501	501	5012

**Table 2:** Experimental environment details.

Experimental Environment	Details
Programming language	Python 3.10
Operating system	Linux, Ubuntu 22.04.5 LTS
Deep learning framework	PyTorch 2.4.1, CUDA 11.8
GPU	NVIDIA RTX 3090 Ti (NVIDIA Corporation, Santa Clara, CA, USA)

**Table 3:** Training settings and hyperparameters used in the experiments.

Parameter	Setting	Parameter	Setting
Input image size	640×640 pixels	Target category	Smoke
Maximum training epochs	300	Batch size	8
Dataloader workers	4	Optimizer	AdamW
Learning rate	0.01	Momentum	0.937
Weight decay	0.0005	Warm-up epochs	3
Warm-up momentum	0.8	Early stopping patience	50 epochs
Automatic mixed precision	Disabled		

**Table 4:** Ablation results of LRM-YOLO on the UFFS dataset.

Model	RepViTBlock	LECD	MPDIoU	Precision/%	Recall/%	mAP50/%	mAP50-95/%	Params/M	GFLOPs	FPS
YOLOv10n	×	×	×	89.2	86.9	93.8	64.5	2.691	8.22	357
+ A	✓	×	×	92.2	85.1	94.1	65.3	2.423	7.64	455
+ B	×	✓	×	91.6	90.1	94.8	67.4	1.701	4.72	833
+ C	×	×	✓	89.6	88.4	94.1	64.7	2.691	8.22	357
+ A + B	✓	✓	×	92.6	89.3	94.8	67.7	1.704	4.74	714
+ A + C	✓	×	✓	92.6	88.2	94.0	65.3	2.423	7.64	455
+ B + C	×	✓	✓	91.8	87.1	95.0	67.9	1.701	4.72	833
+ A + B + C	✓	✓	✓	92.8	90.1	95.1	68.2	1.704	4.74	714

A: RepViTBlock; B: LECD; C: MPDIoU. ✓ indicates that the corresponding module is included, whereas × indicates that it is not included.

**Table 5:** Ablation experiments on different attention mechanisms in MBConv.

Attention	Precision/%	Recall/%	mAP50/%	mAP50-95/%	Params/M	GFLOPs
None	89.6	88.3	94.0	66.3	1.593	4.61
SE	90.4	89.4	93.9	66.1	1.614	4.42
CBAM (Ours)	91.6	90.1	94.8	67.4	1.701	4.72

**Table 6:** Comparison of Detection Performance Among Different Models on the UFFS Dataset.

Model	Precision/%	Recall/%	mAP50/%	mAP50-95/%	Params/M	GFLOPs	FPS
YOLOv10n (Baseline)	89.2	86.9	93.8	64.5	2.691	8.22	357
YOLOv5n	90.2	89.1	94.1	66.5	2.185	9.03	769
YOLOv6	85.7	87.2	92.3	62.8	5.603	12.14	526
YOLOv7-tiny	87.9	88.5	91.7	64.2	6.052	23.78	555
YOLOv8n	90.2	90.0	94.7	67.8	2.684	6.83	769
YOLOv10s	89.8	88.3	93.3	65.0	8.025	24.38	526
YOLOv11n	90.8	89.1	94.6	65.6	2.581	6.34	833
YOLOv12n	89.3	84.9	92.3	63.1	2.502	5.76	714
YOLOv13n	92.4	89.7	94.8	65.8	2.441	6.21	384
YOLO26n	90.5	89.1	94.2	66.8	2.364	5.24	909
LRM-YOLO (Ours)	92.8	90.1	95.1	68.2	1.704	4.74	714

**Table 7:** Cross-Dataset Validation on the Wildfire Smoke V1 Dataset.

Model	Precision/%	Recall/%	mAP50/%	mAP50-95/%	Params/M	GFLOPs	FPS
YOLOv10n (Baseline)	74.6	63.3	71.8	32.8	2.691	8.22	357
LRM-YOLO (Ours)	75.6	72.8	77.9	34.5	1.704	4.74	714

**Table 8:** Composition of the challenging positive small-smoke subset with target areas below 1% of the image area.

Sample Category	Number of Images	Proportion (%)
Small smoke with cloudy backgrounds	49	27.53
Small smoke under backlighting	69	38.76
Thin or visually weak small smoke	60	33.71
Total	178	100.00

**Table 9:** Comparison of YOLOv10n and LRM-YOLO on the challenging small-smoke subset of the UFFS test set.

Model	Images	GT	TP	FN	FP	Precision@Conf0.25 (%)	Recall@IoU0.5 (%)	F1-Score (%)	Miss Rate (%)	FPPI	mAP50 (%)
YOLOv10n	178	178	166	12	28	85.57	93.26	89.25	6.74	0.157	95.19
LRM-YOLO	178	178	171	7	22	88.60	96.07	92.18	3.93	0.124	96.30

## Data Availability

The data presented in this study are available upon reasonable request from the corresponding author. The self-constructed UFFS dataset is available from the corresponding author upon reasonable request. The public Wildfire Smoke V1 dataset used for external validation is publicly available at https://github.com/aiformankind/wildfire-smoke-dataset (accessed on 13 February 2025).

## Abbreviations

The following abbreviations are used in this manuscript:

AFN	Adversarial Fusion Network
CBAM	Convolutional Block Attention Module
CIoU	Complete Intersection over Union
CNN	Convolutional Neural Network
DW	Depthwise Convolution
Faster R-CNN	Faster Region-based Convolutional Neural Network
FN	False Negative
FP	False Positive
GFLOPs	Giga Floating-Point Operations
GELU	Gaussian Error Linear Unit
Grad-CAM	Gradient-weighted Class Activation Mapping
HSV	Hue, Saturation, and Value
LECD	Lightweight Efficient Convolutional Detection head
LRM	LECD, RepViTBlock, and MPDIoU (initial letters of the three core modules)
mAP	Mean Average Precision
mAP50	Mean Average Precision at IoU = 0.5
mAP50-95	Mean Average Precision averaged over IoU thresholds from 0.5 to 0.95
MBConv	Mobile Inverted Bottleneck Convolution
Mixed-NMS	Mixed Non-Maximum Suppression
PW	Pointwise Convolution
R-CNN	Region-based Convolutional Neural Network
RepViTBlock	Re-parameterized Vision Transformer Block
RT-DETR	Real-Time Detection Transformer
SE	Squeeze-and-Excitation
SPPF	Spatial Pyramid Pooling-Fast
SSD	Single Shot MultiBox Detector
SVM	Support Vector Machine
TP	True Positive
UAV	Unmanned Aerial Vehicle
YOLO	You Only Look Once
